# Paralumbar compartment syndrome, a rare sequela of deadlifting: a case report and review of current literature

**DOI:** 10.1186/s13018-024-04860-3

**Published:** 2024-06-23

**Authors:** Mark LaGreca, Thomas Falconiero, Anthony Viola, Aakash Patel, Arash Bagragheh, Brian Danshaw, Scott Rushton

**Affiliations:** 1 Department of Orthopedic Surgery, Philadelphia College of Osteopathic Medicine, 4190 City Ave, Suite 409, Philadelphia, PA 19131 USA; 2 Department of Medicine, Philadelphia College of Osteopathic Medicine, 4190 City Ave, Suite 100, Philadelphia, PA 19131 USA; 3 Main Line Health Orthopaedics & Spine, Wynnewood, PA 19096 USA

**Keywords:** Paralumbar compartment syndrome, Low back pain, Fasciotomies Weight Lifting

## Abstract

**Background:**

Compartment syndrome is a well-known phenomenon that is most commonly reported in the extremities. However, paralumbar compartment syndrome is rarely described in available literature. The authors present a case of paralumbar compartment syndrome after high intensity deadlifting.

**Case presentation:**

53-year-old male who presented with progressively worsening low back pain and paresthesias one day after high-intensity deadlifting. Laboratory testing found the patient to be in rhabdomyolysis; he was admitted for intravenous fluid resuscitation and pain control. Orthopedics was consulted, and Magnetic Resonance Imaging revealed significant paravertebral edema and loss of muscle striation. Given the patient’s lack of improvement with intravenous and oral pain control, clinical and radiographic findings, there was significant concern for acute paralumbar compartment syndrome. The patient subsequently underwent urgent fasciotomy of bilateral paralumbar musculature with delayed closure.

**Conclusion:**

Given the paucity of literature on paralumbar compartment syndrome, the authors’ goal is to promote awareness of the diagnosis, as it should be included in the differential diagnosis of intractable back pain after high exertional exercise. The current literature suggests that operative cases of paralumbar compartment syndromes have a higher rate of return to pre-operative function compared to those treated non-operatively. This case report further supports this notion. The authors recommend further study into this phenomenon, given its potential to result in persistent chronic exertional pain and irreversible tissue damage.

## Introduction

Paralumbar compartment syndrome is a rare condition that is frequently overlooked during initial presentation due to its nonspecific characteristics that can resemble more common conditions. It was first described by Carr et al. who also did an anatomy dissection and coined the term ‘paralumbar compartment syndrome' [1]. In this manuscript, the authors detail a case of paralumbar compartment syndrome with a review of the literature. Our patient, a 53-year-old male, exhibited progressively worsening lower back pain and paresthesias one day after engaging in high-intensity deadlifting.

### Case presentation

The patient is a 53-year-old male who presented with progressively worsening lower back and upper buttock pain with paralumbar paresthesias for about 24 h prior to presentation. The patient performed heavy deadlifting the day prior. He denied any bowel or bladder dysfunction, direct trauma to the area, history of back surgery, history of similar symptoms, radiation of pain, radicular symptoms, or lower extremity numbness or weakness. He denied intravenous drug abuse or other recent drug abuse. He has a past medical history including bipolar disorder, anxiety, depression and hypercholesterolemia.

In the emergency department (ED) he was hemodynamically stable and afebrile. Physical examination was significant for intense paraspinal muscle prominence bilaterally with diffuse tenderness to palpation along the paraspinal muscles. He reported subjective paresthesias and subjective sensory loss to the overlying skin of the lumbosacral region paravertebrally. The midline lumbar spine was minimally tender to palpation, posture was neutral, motor strength and sensation were intact distally. Patient was noted to be standing in the examination room. Table 1 summarizes the results of laboratory studies, which showed elevated blood urea nitrogen (BUN)/creatinine (Cr) ratio, creatine kinase (CK), alanine transaminase (ALT), and aspartate transaminase (AST), and white blood cell (WBC) count. Urinalysis including drug screen was positive for hematuria, ethanol, cannabinoids and opiates.

**Table 1:** Laboratory values for the patient among presentation to the emergency department

Laboratory test	Value	Normal range
BUN/Cr	18/1.4	10/1–20/1
AST	562 IU/L	14–40 IU/L
ALT	112 IU/L	15–58 IU/L
CK	76,133 IU/L	< 190 IU/L
WBC	16.62 × 10^9/L	4.5 to 11.0 × 10^9/L

Labs notable for: BUN/Cr 18/1.4 (baseline 1.1); AST 562; ALT 112; CK 76,133; WBC 16.62; H/H 15.9/44.7; Urine Drug Screen: (+) Ethanol, (+) cannabinoids, (+) opiates; ( +) Hematuria.

**Fig. 1:**
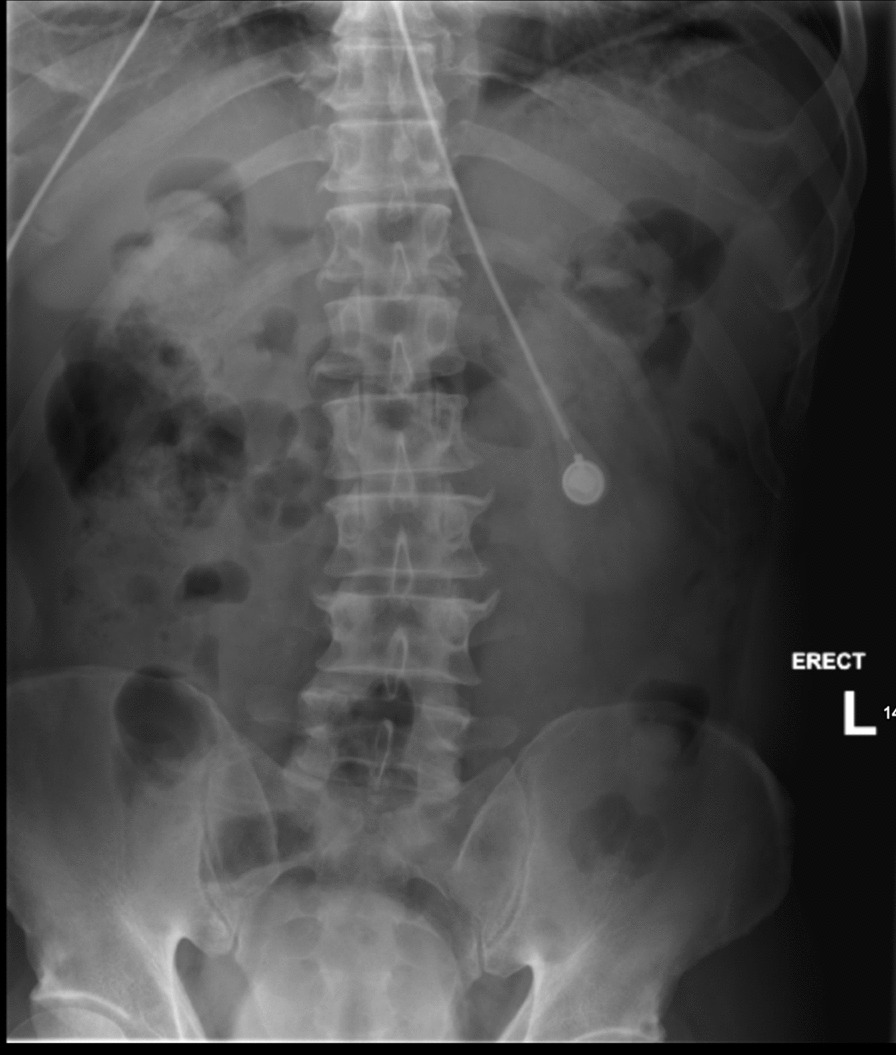
Anteroposterior X-ray of lumbar spine demonstrating no fractures or dislocations

**Fig. 2:**
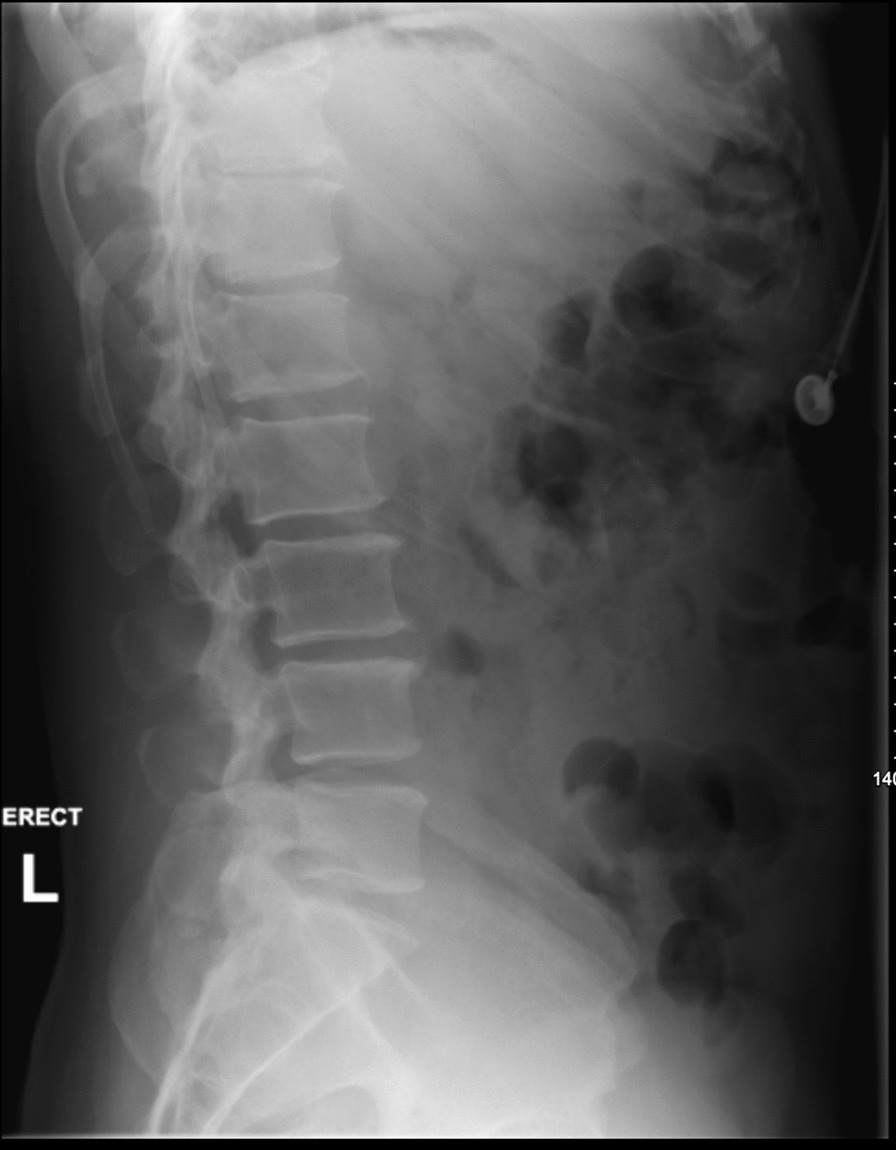
Lateral X-ray image of the lumbar spine demonstrating straightening of the lumbar lordosis, otherwise no fractures or dislocations

Lumbar spine X-rays were obtained in the ED and were unremarkable for fracture or dislocation but were notable for straightening of the normal lumbar lordosis, with mild degenerative changes at multiple levels. This can be seen in Figs. 1 and 2**.**

The patient was subsequently admitted for rhabdomyolysis and acute kidney injury. Initially, the patient received intravenous (IV) and oral pain medication, and resuscitative IV fluids to address the rhabdomyolysis. Orthopedics were consulted the following morning due to persistent lower back pain despite the multi-modal pain regimen administered.

**Fig. 3:**
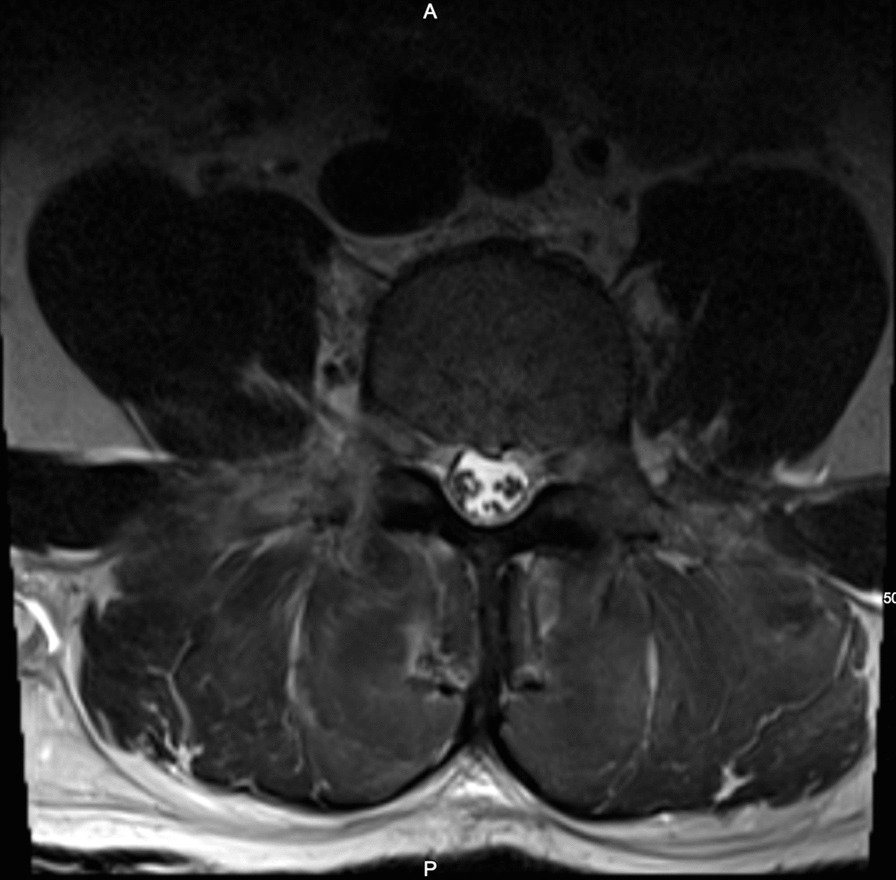
T2 Weighted Image axial cut of the lumbar spine demonstrating severe paralumbar edema and loss of muscle striations. No cord compression noted

**Fig. 4:**
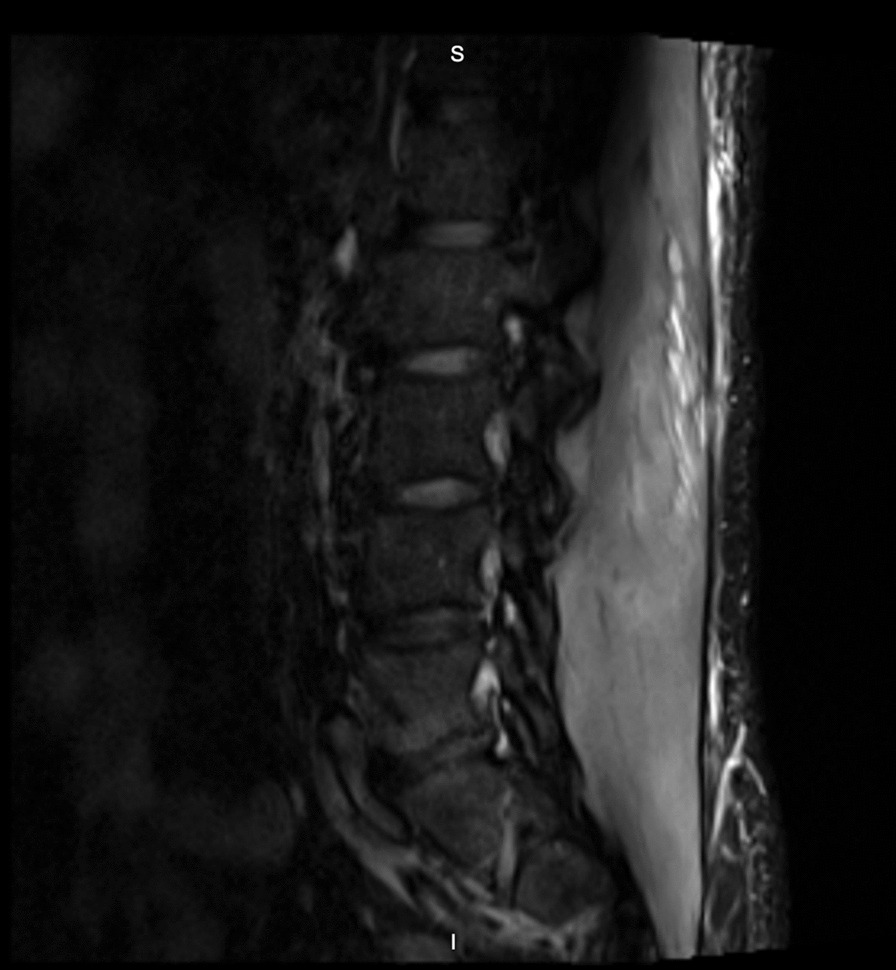
Sagittal STIR MR image of the lumbar spine demonstrating profound paralumbar edema

The patient underwent examination by the on-call orthopedic surgery resident, with no significant changes noted in the examination findings at that time. Lumbar spine magnetic resonance imaging (MRI) was ordered and obtained later the same day. It was reviewed by the facility’s attending spine surgeon and was read as ‘dramatic paraspinal muscle enlargement edema and loss of signal characteristics with a homogeneous appearance of the multifidus, erector spinae extending from the L1 to down to and including the L5/S1 lumbosacral junction’. Also noted on the MRI was significant enlargement of the paraspinal musculature with loss of striations within the muscle compartment, no associated nerve root or spinal canal compromise, and no associated disc pathology with the exception of age-related disc degenerative changes. The diagnosis of paralumbar compartment syndrome was confirmed based on clinical symptoms, presentation and MRI findings, without the need for intracompartmental pressures. MR images can be seen in Figs. 3 and 4.

Following the attending spine surgeon's review of the MRI findings, a discussion was had with the patient with the strong recommendation of urgent surgical decompression. Given the patient’s increasing amount of narcotics to control pain and significant MRI findings, the patient was agreeable to undergo bilateral staged paraspinal fasciotomies. The patient was taken back to the operating room about 36 h after presentation to the ED.

### Stage 1 bilateral fasciotomy

Incisions were marked paraspinal approximately 2–1/2 to 3 fingerbreadths off the midline. Vertical incisions were marked and infiltrated with a dilute solution of epinephrine. Incisions were carried down bilaterally in the paraspinal region out over the lateral musculature. The subcutaneous tissue was mobilized, allowing access to the paraspinal fascia.

**Fig. 5:**
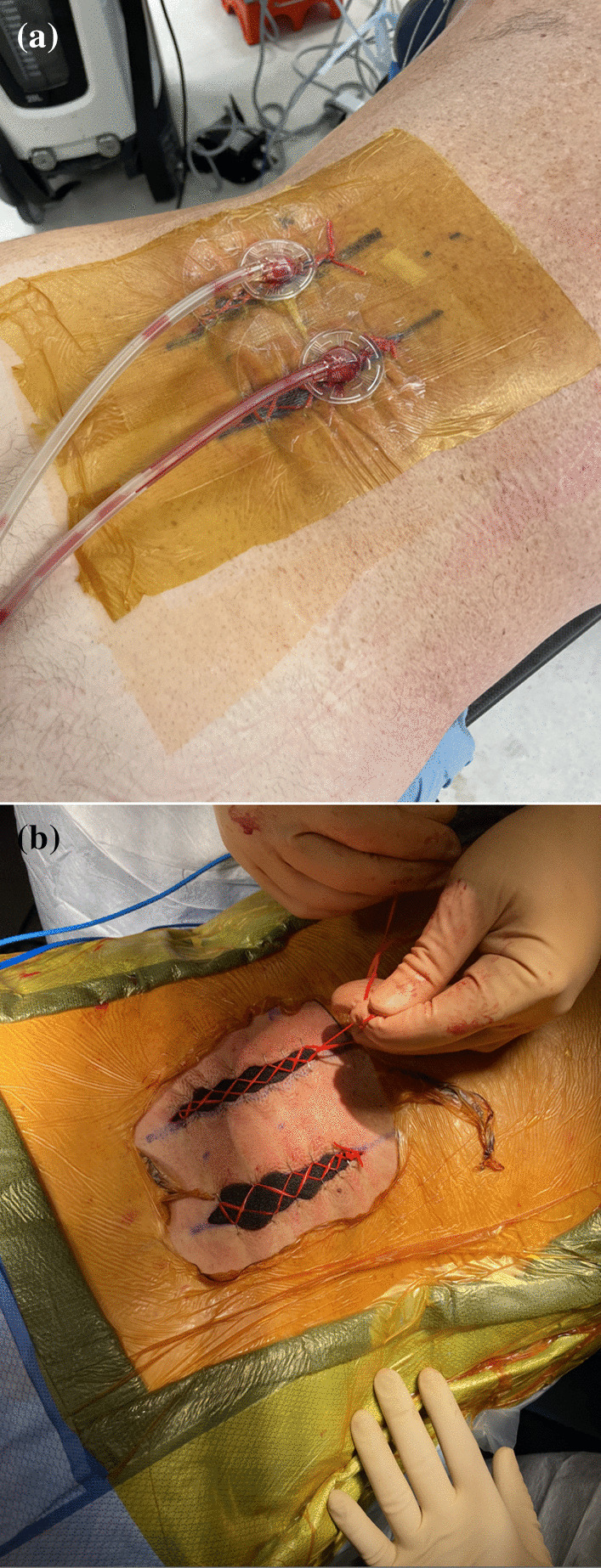
**A** Intra-operative photograph taken after the fasciotomy demonstrating Jacob’s ladder technique with wound vac application. **B** Intra-operative photograph taken after the fasciotomy demonstrating Jacob’s ladder technique with wound vac application

Intraoperative findings included tense and rigid paraspinal muscle mass. Using a 15-blade to avoid further muscle trauma, the fascial layer was released in a vertical fashion. Immediate extravasation of muscle through the fascial defect was encountered, consistent and definitively confirming the presurgical diagnosis. The muscle tissue appeared ashen, but upon release, regained good turgor and vascularity. Paraspinal release included the erector spinae and the more medial compartment along the multifidus. The fascial incision was tracked subcutaneously both cephalad and caudal down to the level of the iliac crest. This procedure was performed bilaterally and definitive improvement in the turgor, color and vascularity were appreciated upon release. The wound was irrigated with pulse lavage and a vac sponge was placed in both incisions and a railroad vessel loop closure was performed for a planned staged return to the operating room for primary wound closure. This can be seen in Fig. 5A, B.

Immediately postoperatively the patient had immediate pain relief. His output drainage from the wound vac decreased every day prior to definitive closure four days later and the output appeared to be serosanguinous in nature. His narcotic use decreased significantly, and he was able to be weaned to oral pain medication prior to the closure of the wounds. He was subsequently taken back to the operating room for wound vac removal and definitive primary closure of the fasciotomy wounds four days later.

### Closure of fasciotomy wounds

The wound vac was removed, including the removal of the tissue expander initially on the right and then the contralateral side on the left. The wound was irrigated bilaterally with one liter of irrigation delivered to the right and then the left incision. The incision was debrided, including freshening of the skin edges, subcutaneous tissue, and fascia. The muscle had punctate bleeding and appeared well vascularized. Vancomycin powder was placed in both incisions. The right incision was closed initially using a 2–0 Vicryl suture followed by staples. A contralateral incision on the left, 2–0 Vicryl, followed by staples.

The patient thereafter had an uneventful postoperative course and was discharged home within one day of the second procedure.

The patient followed up in the office roughly 10 days after the second procedure for a wound check. He stated he had been more active the past two or three days by increasing his walking. The patient had noticed some increased swelling around the left paraspinal incision but no fevers, chills, or excessive drainage from the incision. Otherwise, the patient had no other complaints and had been progressing well. On examination, he demonstrated 5/5 strength in the bilateral lower extremities, with sensation intact to light touch bilaterally to the L3-S1 dermatomes. The incisions were healing well with staples intact, a fullness was palpated over the left paraspinal incision. Clinically, the swelling appeared to be a seroma. Management options for this seroma were discussed, and the patient agreed to undergo a seroma aspiration. Risks/benefits were explained, including infection and recurrence of seroma.

The left paraspinal region was sterilized in the usual fashion. A 20-gauge needle was introduced into the subcutaneous region next to the left paraspinal incision. Forty cc of serosanguineous fluid was aspirated.

He felt significantly better after the aspiration. The patient was instructed to watch for signs of redness, drainage, fever, and chills. He was instructed to return to the office in one week for staple removal and a repeat wound check. The patient returned to the office in one week and was doing well overall. He had a slight recurrence of the seroma but no fevers, change in pain, constitutional complaints, or drainage. On examination, incisions were well healed, left-sided incision showing a seroma, no drainage or erythema. Suture lines were intact. He remained motor and neurovascularly intact.

The patient was instructed to follow up in two weeks for another clinical re-evaluation; however, he canceled his appointment since he was feeling well. The patient was instructed to follow up as needed in the office, but he has not followed up since. This case report was written about 30 months after this case was done without any more follow up. A brief phone call was had with the patient for consent to publish the findings of this case report. The patient reported that he had no functional limitations or pain at this time. He was back to performing all activities of daily living including weight lifting.

## Discussion

Paralumbar compartment syndrome has been reported in the literature very infrequently. To date, there are no level 1 studies or strong recommendations for the treatment of paralumbar compartment syndrome. According to a recent systematic review, paralumbar compartment syndrome is common after patients participated in some sort of strenuous exercise, specifically high-intensity deadlifting—almost 50% of all case reports [2]. Patients diagnosed with paralumbar compartment syndrome appeared to present with similar symptoms, including low back pain with a focus of subjective decreased sensation over the lumbosacral region. On examination, the patients were found to have ‘rigid’ or ‘board-like’ paraspinal musculature without any motor or neurological symptoms. These patients tend to be admitted for rhabdomyolysis or acute kidney injury and were initially treated with IV fluids, urine alkalization, and IV narcotic pain medication, with escalating narcotic requirements over time. Magnetic resonance imaging studies were obtained at some point during the patients’ hospital stay, which shows a characteristic ‘extensive edema in the paraspinal musculature’ [2]. In light of these symptoms and findings, it is imperative for physicians to recognize the possibility of paralumbar compartment syndrome as a differential and promptly engage in consulting orthopedic surgery. Delayed intervention is shown to lead to undiagnosed or delayed diagnosis of paralumbar compartment syndrome, and this may lead to significant and chronic disability, impacting the patient's daily life activities.

Both of the cases reported by Haig et al. exemplifies the diagnostic challenges associated with paralumbar compartment syndrome [3]. In their initial case, they describe a patient who underwent aortoiliac bypass surgery. Following the procedure, the patient developed persistent low back pain and subsequently underwent an MRI, which was read as a slight disc herniation. However, over a 10-month period, the patient's symptoms persisted, presenting as ongoing low back pain exacerbated by daily activities and accompanied by lumbar region paresthesia. Subsequent MRI scans conducted 11 months post-surgery revealed distinctive features, including atrophic left paraspinal musculature with fatty replacement and a cranial–caudal distribution of signal change suggestive of muscle degeneration and fatty infiltration. These MRI findings were indicative of a likely missed paralumbar compartment syndrome which had a significant effect on this patient’s day to day life.

Ilyas et al. recently published a systematic review comparing outcomes of the reported case reports of paraspinal compartment syndrome and found that despite the variable follow-up timing for these patients, most patients returned to pre-operative function with operative treatment [2]. One common theme found, including in this case report, that the patient felt immediate pain relief post-operatively. It was noted in this systematic review that patients who were not treated operatively experienced mild intermittent back pain associated with exertion post-operatively [2]. This could be devastating in patients who are very physically active. Based on this case report and similar findings, operative intervention is recommended in the acute setting, offering positive outcomes and symptom relief. Notably, the patient in this case opted for operative treatment and experienced significant improvement, as evidenced by the cancellation of his four-week post-operative visit due to improved well-being.

Given the rarity of this condition in the literature, the authors presented this case in order to spread awareness of this diagnosis, especially because many of the case reports written had significant lag times to diagnosis [1, 4–8]. The lag time in diagnosis ultimately changed the treatment for some patients. Some non-operative case reports note that the timing and “clinical and laboratory improvement” of the patient led to treating that particular patient conservatively without operative intervention [9, 10]. The majority of cases treated operatively had ‘good’ results with minimal issues in the limited post-op follow-ups reported. This case report furthers this notion.

## Conclusion

Paraspinal compartment syndrome is a rare phenomenon found in patients with a recent history of strenuous high-intensity exercise presenting with severe low back pain, subjective sensory changes in the region of the pain, exam revealing rigid/tense paraspinal musculature without motor or neurological findings and advancing imaging such as MRI revealing extensive edema in the paraspinal musculature. Based on the limited amount of literature that is currently available, if caught early enough, paraspinal compartment syndrome should be treated with fasciotomies to prevent long-term sequelae of chronic back pain, specifically with exertion. Further research should be conducted into this phenomenon with appropriate follow-up and standardized patient pain/function scales for back pain.

## Data Availability

No datasets were generated or analysed during the current study.

## Abbreviations

MRI: Magnetic resonance imaging
ED: Emergency department
BUN: Blood urea nitrogen
Cr: Creatinine
CK: Creatine kinase
ALT: Alanine transaminase
AST: Aspartate transaminase
WBC: White blood cell
